# Generation Rx: evaluation of medication misuse prevention education for older adults

**DOI:** 10.3389/fpubh.2024.1441904

**Published:** 2024-09-12

**Authors:** Ruth E. Emptage, Brittany S. Lovatt, Cynthia D. Sloan, Junan Li, Molly N. Downing, Katherine E. Summers

**Affiliations:** College of Pharmacy, The Ohio State University, Columbus, OH, United States

**Keywords:** drug misuse, medication safety, prevention education, evaluation, older adult

## Abstract

**Objective:**

Increased medication misuse over the last two decades has prompted extensive discussion about the lack of evidence-based and evidence-informed prevention education programs targeting the topic. As older adults are high utilizers of medications, this is an important population to reach with such educational programming. This study was designed to assess the change in knowledge and behavioral intentions of older adult participants after attending an educational session focused on safe medication use utilizing the Generation Rx Older Adult Toolkit (GROAT) resources.

**Methods:**

The Generation Rx team at The Ohio State University College of Pharmacy (OSU COP) partnered with The Ohio State University Extension offices (OSU Extension) across the state of Ohio to provide GROAT educational programming in their communities. OSU Extension Educators were trained via the standardized virtual training program, *Generation Rx Ambassadors*. Program participants were surveyed immediately before and after the educational events. Pre- and post-survey data was then analyzed to assess knowledge gain and behavioral intentions about safe medication practices, as well as program perception and program satisfaction.

**Results:**

Programming occurred between May 2022 and September 2022. In total, OSU Extension Educators collectively engaged 843 individuals in a prevention education program utilizing the GROAT materials. After excluding participants under 50 years of age, there were 297 pre surveys and 245 post surveys included in the data analysis. Knowledge gains from pre- to post-survey showed a significant increase in correct responses in seven of the eight questions asked regarding safe medication practices. All five questions evaluating behavioral intentions demonstrated positive results after the programming (*p* < 0.001). Participants’ perceptions and program satisfaction were also favorable.

**Conclusion:**

This study found through pre- and post-survey results that the Generation Rx Older Adult Toolkit programming delivered by Generation Rx trained OSU Extension Educators significantly increased older adult participants’ knowledge and favorably impacted behavioral intentions around safe medication use practices.

## Introduction

The rise in prescription medication use across the United States is well documented—longer lifespans, increasing rates of chronic disease, and growing reliance on prescription medications all contribute to the trend (1). Greater prescription use increases the potential for prescription drug misuse, which includes using more medication than prescribed, using a medication for a reason other than prescribed, or sharing and/or using someone else’s medication. In fact, nearly 51% of those who misuse prescription pain relievers obtain them from family or friends per the 2019 National Surveys on Drug Use and Health (2). There has been significant public discussion about the linkage between the misuse of prescription pain relievers and the opioid crisis in recent years. The misuse potential of prescription stimulants and sedatives, however, has been less emphasized, despite the increase in their use. In an analysis of modern drug trends, Ho found that both men and women are consuming large quantities of prescription medication at the same time, for longer periods, and for a growing number of health concerns (1). It was also noted that 52% of men and 62% of women in the population were using prescription drugs in 2019. Increased prescription drug misuse in the 21^st^ century has prompted extensive discussion about the availability of evidence-based and evidence-informed prevention education programs to address the topic (3–5).

Although most individuals will take medicine at some point in their lives, formal training on safe medication-taking behaviors (e.g., in K-12 settings, independent senior living settings, etc.) has rarely been introduced or maintained in local communities. Implementing best-practice prevention education is a critical response to this concern. Significant investment by federal and state health entities have targeted specific populations, particularly youth audiences; however, an equivalent level of investment for the older adult population has not occurred, despite its growing numbers. According to the US Census Bureau, there were over 99 million people over the age of 55 living in the United States between 2020–22 (6). Schepsis and colleagues, utilizing the National Surveys on Drug Use and Health data, found prescription drug misuse increased in those over age 50 when comparing survey results from the years 2002–2003 to 2012–2013. When publishing their results, the authors called for public health and educational efforts to target this increase in medication misuse by older adults (7).

When working with older adult populations, it is critical that educational plans recognize the unique medication taking experience that characterizes this period of life. For example, an adult 60 years of age likely serves in several roles: he or she may be a caregiver to young people, use medication therapies in his/her daily routine, and/or be the caregiver to an older adult or parent. More recent studies have concluded those 65 years of age or greater accounted for the highest rates of polypharmacy (the use of five or more drugs concurrently) (1). Thus, this segment of the population is often responsible for managing a higher quantity of prescription drugs—including the safe storage and disposal of those drugs—than younger members of their communities. In a review of effective prevention strategies, Griffin and Botvin noted that adults can have a positive or negative impact on the family factors that contribute to youth substance misuse (3). Since older adults often serve as head-of-families, they are positioned to influence matters of health and well-being among their family members based on their own medication experiences and knowledge (8).

Effective educational programming must emphasize safe medication practices. If the education is effective, older adults will fully understand their responsibilities as their own health advocate, their role as a potential caretaker/mentor, and the importance of safe storage and disposal of medications. For these reasons, a successful approach must consider multiple community settings, i.e., senior centers, faith centers, etc., to reach this cross section of the population. Generation Rx (GenRx) was established in 2007 at The Ohio State University College of Pharmacy (OSU COP) to teach safe medication-taking practices across the lifespan. This research’s objective is to assess the change in knowledge and behavioral intentions of older participants after attending an educational session focused on safe medication use utilizing the Generation Rx Older Adult Toolkit (GROAT) resources. Designed by an interdisciplinary team of pharmacists, health educators, and community stakeholders, the program incorporates best-practice resources and a sustainable training and delivery approach to empower communities in prescription drug misuse prevention (9). Thus, drawing on the best practices in education and prevention sciences noted here, GenRx faculty and staff developed the GROAT content (10) for dissemination in community-based settings supporting the older adult population. These materials have been widely used since becoming freely available in 2018.

Since the professionals who work for The Ohio State University Extension (OSU Extension) share a mutual interest in public health and have the local presence and relationships needed to support prevention, the GenRx team partnered with OSU Extension to provide GROAT programming across the state of Ohio. This research evaluates the effectiveness of GROAT programming using this delivery model. This is the first evaluation of the GROAT content by the target audience. Establishing the effectiveness of these materials would strengthen its usefulness as a standardized tool in delivering safe medication use practices to older adults.

## Materials and methods

### Intervention and implementation

GenRx faculty and staff partnered with OSU Extension to deploy GROAT programming in Ohio. OSU Extension maintains offices in every county and sustains relationships with community-based organizations that serve the targeted audience. Pre- and post-intervention surveys previously developed and piloted were provided to program participants by the OSU Extension Educators.

Programming and survey collection began on May 2, 2022, and continued to September 30, 2022. OSU Extension offices in twelve Ohio counties received grant funds to participate in the project. All OSU Extension offices were required to appoint an OSU Extension Educator from the Family and Consumer Sciences department to coordinate the local efforts. Each of the selected educators completed the virtual training program, *Generation Rx Ambassadors* (11) to standardize delivery of GROAT programming. They were trained in the project’s evaluation protocols and were responsible for coordinating the local programs and maintaining program fidelity in various community-based settings. OSU Extension Educators received funds to support program implementation efforts including printing, giveaways, and marketing materials. Eight of the twelve counties successfully reached their participant goals and collected survey data. The GenRx team, along with the OSU Extension Educators successfully implemented 56 community programs, with an average of fourteen individuals per session (Table 1). Each session began with a pre-survey completed by participants, proceeded with either a slide-based presentation or trivia game activity from the GROAT materials, and concluded with a post-survey. The locations utilized for the sessions varied between counties and included senior centers, libraries, older adult independent living facilities, faith centers and community centers.

**Table 1:** OSU extension programming sites by Ohio County.

Ohio County	Number of educators trained	Number of events offered	Number of pre-surveys *n* (%)	Number of post-surveys *n* (%)
Carroll	1	5	35 (11.8%)	34 (13.9%)
Darke	1	9	13 (4.4%)	15 (6.1%)
Franklin	3	10	68 (22.9%)	41 (16.7%)
Miami	1	1	16 (5.4%)	6 (2. 5%)
Montgomery	7	12	44 (14.8%)	37 (15.1%)
Pike	1	4	34 (11.4%)	28 (11.4%)
Warren	1	7	31 (10.4%)	33 (13.5%)
Wood	1	8	54 (18.2%)	50 (20.4%)
Number of surveys missing county information			2 (0.7%)	1 (0.4%)
Total	16	56	297	245

The GROAT programming is manualized with clear facilitator guides and program materials. This approach ensured every participant received a similar program experience despite varying program facilitators. Additionally, the research team provided training, guidance, and clear procedures to all the local site leaders. Each OSU Extension Educator was instructed on data collection and program delivery standards to avoid confounding variables and threats to internal/external validity, including timeline for when pre and post program data was to be collected and sample programming size requirements.

### Participants

**Figure 1:**
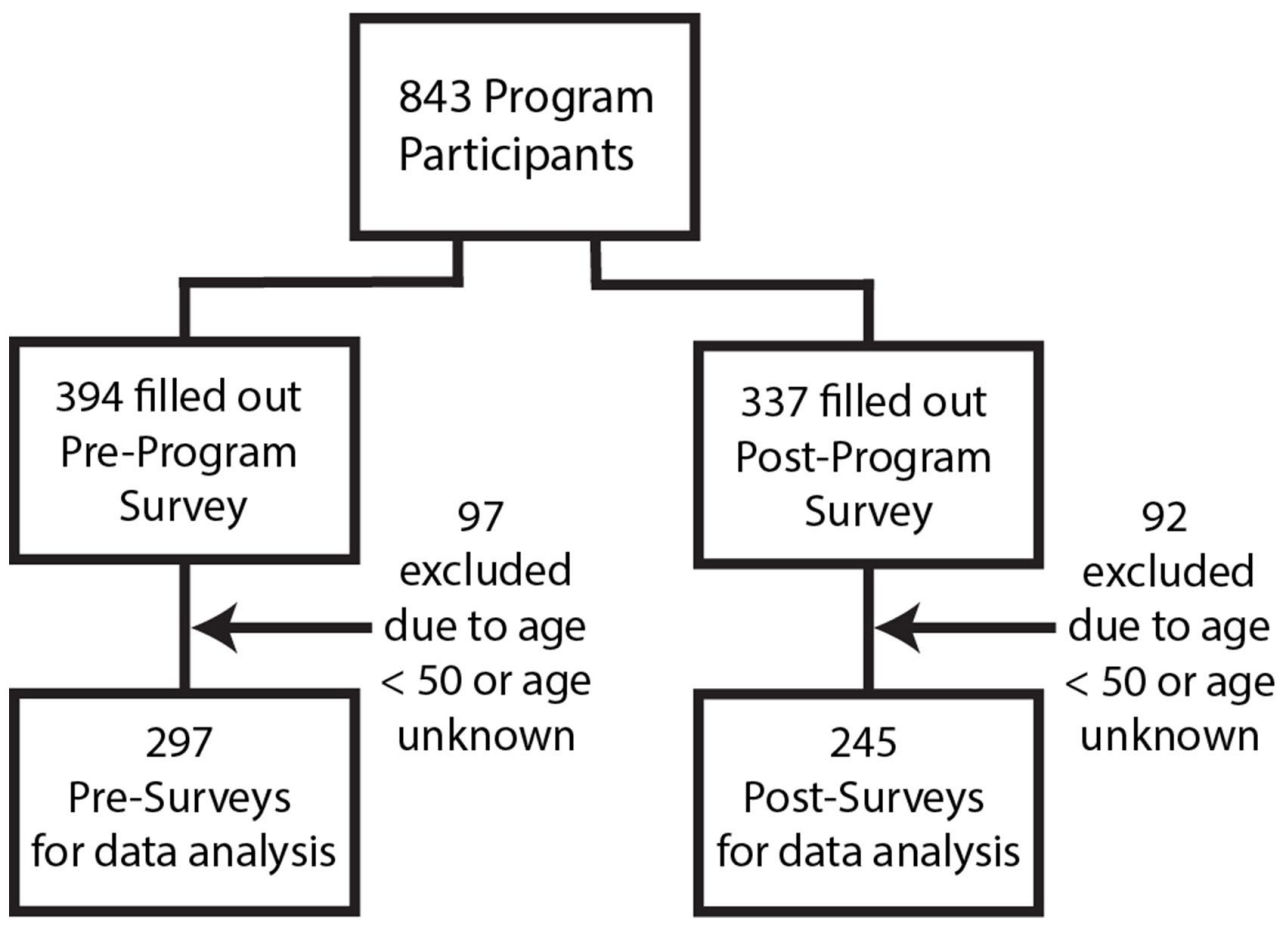
Program participants and survey collection.

The participants in the GROAT educational programming consisted of 843 adults. Participants resided in an urban, suburban, or rural community within Ohio depending on which educational session they attended. Those who completed surveys but were under the age of 50 were excluded. There were 297 participants who completed the pre-survey and 245 who completed the post-survey. Figure 1 shows the numbers of program participants and those included in the data analysis.

### Survey instruments design

The survey instruments were developed by subject matter experts from within the GenRx team and The Ohio State University. The instruments were subsequently evaluated for content validity. Survey questions were designed to assess participant demographics, knowledge gains, behavioral intentions, perceptions, and participant satisfaction. Demographic information was collected via single-select and multi-select multiple choice questions. Survey questions to assess knowledge gains were multiple choice with one or more correct answer responses. Questions were created to assess each of the three learning outcomes of the GROAT educational content. The key messages associated with each learning outcome are be your own health advocate by being informed about the medications that you take, learn and model safe medication practices, and know the risks of medication misuse. The behavioral intentions questions were posed to gather yes or no responses. Participants’ perceptions and satisfaction with the programming were gathered on the post-survey using 5 category Likert scale questions.

The survey questions were piloted in multiple counties the year prior to this study (2021) by OSU Extension Educators from a previous grant project. Question performance was evaluated by the research team and questions were adjusted for clarity and brevity. The research team also garnered input directly from the educators who were involved in the pilot use of the surveys for necessary modifications. The surveys were estimated to take 10–15 min to complete.

### Data collection outcome measures

Both pre- and post-intervention surveys were administered immediately before and immediately after the OSU Extension Educators completed the GROAT programming. The surveys were distributed as paper-based surveys and OSU Extension Educators entered the survey data into an electronic pre- or post-survey link. There was also the option to mail the paper surveys to the GenRx research team for electronic entry. The survey data was transferred to a spreadsheet, aggregated, unpaired and prepared for analysis. Demographics, knowledge gains and behavioral intentions were all assessed on both the pre- and post-surveys. Questions to assess participants’ perceptions and satisfaction with the GROAT programming delivered by the OSU Extension Educators were included only in the post-survey.

### Data analysis

The pre-post data was analyzed through a mixed method design to evaluate the achievement of the GROAT key messages by program participants. Descriptive statistics were first used to analyze the data. While categorical data were presented as count (*n*) and frequency (%), Likert scale data (post-intervention survey program perceptions and satisfaction) was summarized as mean (standard deviation). Categorical data were then compared using *χ*^2^ or Fisher’s exact tests where appropriate. Significance level was set at *α* = 0.05 and missing data was not included in statistical comparisons. R3.4 software (The R program for statistical computing, https://www.r-project.org) was used in this study.

## Results

### Sociodemographic characteristics

Of the 843 program participants across the state, 297 (35.2%) completed a pre-survey and 245 (29.1%) completed a post-survey. Pre-survey participants and post-survey participants were well matched regarding demographics except for race (Table 2). There were more participants who identified as Asian in the pre-survey group and more participants who identified as native Hawaiian or other Pacific Islander in the post-survey group. Participants were well matched in the categories of prescription and non-prescription medication use and living environment, which was additional demographic information collected.

**Table 2:** Participant demographics.

Variable	Pre-Survey No. (%) *N* = 297	Post-Survey No. (%) *N* = 245	*p* value*
Age, *y*			0.92
50–59	27 (9.1)	27 (11.0)	0.55
60–69	51 (17.2)	42 (17.1)	0.99
70–79	116 (39.1)	94 (38.4)	0.94
80–89	86 (29.0)	71 (29.0)	0.99
90–99	17 (5.7)	11 (4.5)	0.65
Gender identity			0.98
Male	73 (24.6)	59 (24.1)	0.97
Female	221 (74.4)	184 (75.1)	0.93
Other	1 (0.3)	1 (0.4)	0.99
Wish not to answer	1 (0.3)	1 (0.4)	0.99
Race			0.01
African American	41 (13.8)	32 (13.1)	0.90
Asian	14 (4.7)	3 (1.2)	0.025
Native American/Alaskan Native	2 (0.7)	0 (0)	0.50
Native Hawaiian or Other Pacific Islander	0 (0)	6 (2.4)	0.008
White	215 (72.4)	190 (77.6)	0.20
Multi-racial	14 (4.7)	6 (2.4)	0.18
Other	6 (2.0)	4 (1.4)	0.99
Prefer not to answer	2 (0.7)	0 (0)	0.50
Ethnicity			0.37
Hispanic or Latino	6 (2)	4 (1.6)	0.99
Neither Hispanic nor Latino	220 (74.1)	187 (76.3)	0.34
Wish not to answer	39 (13.1)	22 (9.0)	0.17
Living Environment			0.69
Alone	151 (50.8)	134 (54.7)	0.42
With a spouse, partner, roommate or significant other	103 (34.7)	87 (35.5)	0.91
With a caregiver	3 (1.0)	0 (0)	0.26
With an adult child (daughter, son, step-child, etc.)	21 (7.1)	16 (6.5)	0.94
With a spouse, partner, roommate or significant other AND with an adult child (daughter, son, stepchild, etc.)	3 (1.0)	2 (0.8)	0.99
Wish not to answer	1 (0.3)	0 (0)	0.99
Medication Use			
Prescription medications use daily			0.53
0	14 (4.7)	17 (6.9)	0.36
1–4	145 (48.8)	118 (48.2)	0.95
5–9	87 (29.3)	72 (29.4)	0.99
10+	32 (10.8)	26 (10.6)	0.99
Wish not to answer	3 (1.0)	0 (0)	0.26
Non-prescription medications used daily			0.80
0	34 (11.4)	33 (13.5)	0.56
1–4	188 (63.3)	155 (63.3)	0.99
5–9	48 (16.2)	38 (15.5)	0.93
10+	6 (2.0)	6 (2.4)	0.78
Wish not to answer	2 (0.7)	0 (0)	0.50

^*^χ^2^ test or Fisher’s exact test was used where appropriate. The significance level was *α* = 0.05. Missing data was not included in statistical comparisons.

### Changes in knowledge

Survey questions assessing knowledge gains pre- and post-educational intervention are presented in Table 3 with the correct answer choice(s) demarcated by a # symbol. Seven of the eight questions on the surveys showed significant positive change from pre- to post-survey. This knowledge was gained across all three key message areas of educational programming, including becoming knowledgeable about the medications one takes, learning and modeling safe medication practices, as well as being informed about medication misuse.

**Table 3:** Knowledge gain survey question results.

Question and answer choices	Pre-test *n* (%) *N* = 297	Post-test *n* (%) *N* = 245	*p* value*
*(Pre-survey) I updated my medication record in the last..* *(Post-survey) I will update my medication record in the next..*			<0.001
3 months#	149 (50.2%)	164 (66.9%)	<0.001
6 months#	38 (12.8%)	30 (12.2%)	0.95
12 months#	21 (7.1%)	10 (4.1%)	0.19
I do not keep a medication record	62 (20.9%)	23 (9.4%)	<0.001
I do not take medications	13 (4.4%)	6 (2.4%)	0.25
I will not update my medication record (post-only)	0 (0%)	1 (0.4%)	0.45
Missing response	14	11	
*Which of the following is an example of medication misuse?*			0.011
Taking more medication than instructed	25 (8.4%)	12 (4.9%)	0.15
Sharing medication with others	8 (2.7%)	6 (2.4%)	0.99
Taking a medication for a different reason than prescribed	4 (1.3%)	3 (1.2%)	0.99
All of the above#	211 (71.0%)	210 (85.7%)	<0.001
I do not know	30 (10.1%)	10 (4.1%)	0.012
Missing response	19	4	
*Where do most people who misuse prescription drugs get them?*			<0.001
From their doctor	53 (17.8%)	13 (5.3%)	<0.001
From the internet	9 (3.0%)	5 (2.0%)	0.59
From family and friends#	39 (13.1%)	87 (35.3%)	<0.001
All the above	153 (51.5%)	133 (54.3%)	0.58
I do not know	26 (8.8%)	6 (2.4%)	0.002
Missing response	17	1	
*What is important for you to know about the medication(s) you take? (Select all that apply)*			
The best time of day to take the medication#	193 (65.0%)	214 (87.3%)	<0.001
If it is safe to crush or split tablets#	140 (47.1%)	176 (71.8%)	<0.001
Should it be taken with or without food#	171 (57.6%)	205 (83.7%)	<0.001
Common side effects that could occur#	187 (63.0%)	193 (78.8%)	<0.001
What to do if you forget to take a dose#	177 (59.6%)	181 (73.9%)	<0.001
I do not know	30 (10.1%)	2 (0.8%)	<0.001
Missing response	5	4	
*If given the prescription medication bottle pictured below how many tablets would you take per day?* *(Directions on the label read “Take 1 tablet twice daily”)*			0.089
2 tablets per day#	218 (73.4%)	196 (80%)	
All other responses	79 (26.6%)	49 (20%)	
*Which of the following is the best location to store your prescription medications?*			<0.001
Kitchen cabinet, out of reach	101 (34.0%)	48 (19.6%)	<0.001
Kitchen counter	18 (6.1%)	8 (3.3%)	0.16
Bathroom medicine cabinet	58 (19.5%)	5 (2.0%)	<0.001
Lockbox or other locked location#	92 (31.0%)	160 (65.3%)	<0.001
Missing response	28	24	
*True or False: Nonprescription medications, also called Over the Counter (OTC) medications, are required to have standardized medication information on the packaging.*			<0.001
True#	216 (72.7%)	213 (86.9%)	<0.001
False	23 (7.7%)	17 (6.9%)	0.85
I do not know	48 (26.3%)	9 (3.7%)	<0.001
Missing response	10	6	
*Which of the following is an acceptable way to get rid of prescription medication you are no longer taking?*			<0.001
Give to a friend to use	3 (1.0%)	0 (0%)	0.26
Place in a drug disposal box#	240 (80.8%)	238 (97.1%)	<0.001
Keep all prescription medication	10 (3.4%)	0 (0%)	0.0026
Flush the medication down the toilet	35 (12.8%)	3 (1.2%)	<0.001
Missing response	9	4	

# indicates what was considered to be a correct response. ^*^χ^2^ test or Fisher’s exact test was used where appropriate. The significance level was *α* = 0.05. Missing data was not included in statistical comparisons.

### Changes in behavior

All five survey questions pertaining to behavior change demonstrated significant positive results (*p* < 0.001) as found in Table 4. From pre- to post-survey, a substantial percent of participants stated they would keep an updated list of medications (71.4 to 93.5%), be willing to ask a pharmacist about possible drug interactions when using non-prescription products (52.9 to 88.2%), share their medication record with their healthcare provider at every health care visit (79.1 to 93.5%), dispose of prescription medications when no longer needing them (69.4 to 89.4%), and share the educational materials with others (69.4 to 84.9%).

**Table 4:** Behavioral intention survey question responses.

Survey directions: Please share what you typically do or do not do (pre) or intend to do or not do (post) from the list below:			
Survey question			*p* value*
Pre: I keep an updated, complete record of my prescription and nonprescription medications. Post: I will keep an updated, complete record of my prescription and nonprescription medications.			<0.001
	Pre	Post	
Yes	212 (71.4%)	229 (93.5%)	
No	58 (19.5%)	3 (1.2%)	
Missing response	27	5	
Pre: I ask my pharmacist about possible drug interactions when using non-prescription products. Post: I will ask my pharmacist about possible drug interactions when using non-prescription products.			<0.001
	Pre	Post	
Yes	157 (52.9%)	216 (88.2%)	
No	104 (35.0%)	14 (5.7%)	
Missing response	36	5	
Pre: I share my medication record with my healthcare provider at every health care visit. Post: I will share my medication record with my healthcare provider at every health care visit.			<0.001
	Pre	Post	
Yes	235 (79.1%)	229 (93.5%)	
No	39 (13.1%)	4 (1.6%)	
Missing response	23	5	
Pre: I dispose of prescription medications when I no longer need them. Post: I will dispose of prescription medications when I no longer need them.			<0.001
	Pre	Post	
Yes	206 (69.4%)	219 (89.4%)	
No	53 (17.8%)	4 (1.6%)	
Missing response	38	13	
I will share today’s educational materials with others (family members, teenagers or community members). (Pre and Post)			<0.001
	Pre	Post	
Yes	206 (69.4%)	208 (84.9%)	
No	42 (14.1%)	11 (4.5%)	
Missing response	49	13	

^*^χ^2^ test or Fisher’s exact test was used where appropriate. The significance level was *α* = 0.05. Missing data was not included in statistical comparisons.

### Program satisfaction

Perceptions of the educational programming evaluated on the post-survey are shown in Table 5. When asked, “As a result of today’s session, I am more aware of the importance of taking medications as directed by a healthcare provider,” 233 of 245 (95.1%) total post-survey participants either strongly agreed or agreed. Similarly, when asked if they were more knowledgeable about how to properly read the labels on their prescription and non-prescription medications, 232 (94.7%) respondents answered either strongly agree or agree.

**Table 5:** Post intervention survey program perceptions and satisfaction.

“As a result of today’s session…”	*n* (%) strongly agree (Scale: 5)	*n* (%) agree (Scale: 4)	*n* (%) disagree (Scale: 2)	*n* (%) strongly disagree (Scale: 1)	Mean* (SD)
I am more aware of the importance of taking my medications as directed by a healthcare provider. (*n* = 237)	174 (71.0%)	59 (24.1%)	3 (1.2%)	1 (0.4)	4.70 (0.58)
I am more knowledgeable about how to properly read the labels on my prescription and non-prescription medications. (*n* = 236)	155 (63.3%)	77 (31.4%)	3 (1.2%)	1 (0.4%)	4.62 (0.60)
Please rate your level of agreement with the items in the list below.					
I would recommend this session to a friend or colleague. (*n* = 238)	150 (61.2%)	87 (35.5%)	1 (0.4%)	0 (0%)	4.62 (0.51)
The information gained from today’s session was relevant to me. (*n* = 236)	154 (62.9%)	77 (31.4%)	4 (1.6%)	1 (0.4%)	4.61 (0.63)

^*^Likert scale, strongly agree = 5; agree = 4; disagree = 2; strongly disagree = 1 [used to calculate the Mean and SD (standard deviation)].

An evaluation of satisfaction with the GROAT programming was also conducted. When asked if respondents would recommend the session to a friend or colleague, 237 (96.7%) either strongly agreed or agreed. Finally, 231 (94.3%) strongly agreed or agreed that the information gained from the session was relevant to them.

## Discussion

The evaluation of the medication misuse prevention education using the GROAT materials delivered by OSU Extension Educators demonstrated exceptionally positive results. The educational interventions using the GROAT materials led to knowledge gains in each of the key message areas. The survey questions pertaining to behavioral intentions also demonstrated favorable results in each category assessed. In addition, program participants had favorable perceptions and satisfaction with the educational sessions. These results, if put into action, could lower the risk for older individuals’ experiencing potential medication-related problems and aid in the prevention of medication misuse. Such prevention efforts align with the National Institute on Aging stated future research direction of preventing medication misuse in aging populations (12). They specifically address the need for additional studies aimed at alleviating medication problems in older populations.

Targeting an older adult demographic with medication misuse prevention efforts was further validated by the research conducted by Jallow, et al. They documented older adults’ perspectives on their and other health professionals’ roles in medication safety (13). The researchers’ utilized semi-structured interviews of 28 community-dwelling older adults who took five or more prescription medications daily. The interviews utilized twelve questions developed by the research team. The results of the interviews suggest older adults viewed their responsibilities for their own medication safety as self-learning about their medications and securing the medications. More specifically 4 themes identified by the authors included: taking fewer medication, not missing doses, securing medications and learning about medications.

The GROAT materials align and reinforce these perceived roles found by Jallow et al. as well as target community dwelling older adults who take prescription medications. The GROAT presentation as well as the trivia game begin with a focus on being your own health advocate by becoming knowledgeable about the medication you take. The second message is to learn safe medication practices which includes the following 4 principles: only use prescription medication as directed by a health professional, do not share or take someone else’s medication, keep your medications safe through appropriate storage and disposal practices, and model safe medication practices. This alignment with older adults perceived roles in medication safety strengthens the potential impact of the GROAT programming as the content will presumably resonate with the older adult audience and equip them with applicable information.

While there has been research pertaining to other aspects of medication use in older adults (14, 15), only Whittaker et al. evaluated in-person educational sessions pertaining to safe medication use. Whittaker and colleagues compared the effectiveness of an educational intervention on awareness of medication safety and poison prevention resources in older adults with low health literacy via game-based education versus brochure-based information (16). They utilized a pre- and post-intervention survey design and looked at knowledge gains, behavioral intentions as well as knowledge retention with a 30 days follow up survey. With 27 study participants in the game-based group and 26 in the brochure group, correct post intervention responses among those with incorrect baseline responses showed statistical significance regarding use of childproof caps, interpreting a drug facts label, medication list documentation and who to call for advice in the middle of the night. These authors concluded that live education was more effective than the brochure-based-only approach when educating older adults about medication safety.

Building upon the Whittaker study, this GROAT research expanded upon Whittaker et al.’s findings with a much larger sample size and a more diverse target audience. The GROAT study also had an additional focus on medication misuse prevention. Both study designs used live programming. The GROAT programming utilized OSU Extension events and provided a concise handout—developed with limited literacy and visual considerations in mind (e.g., large font and imagery)—to participants to reinforce the educational messaging and encourage them to share the information with others.

There are several strengths to the GROAT study design. The survey instruments were pilot tested through statewide programming the year prior. Feedback was also gathered from the program facilitators (OSU Extension Educators) after the pilot offering and question adjustments were made to shorten both surveys and clarify question wording. In addition, a robust number of surveys were collected from program participants across eight different counties in Ohio including rural, suburban, and urban areas, providing a range in perspectives and demographics.

This research demonstrates the usefulness of the educational content of the GenRx Older Adult Toolkit. The older adult population is a useful target for this education given their high use of medications and their position of influence in their families. Through either a trivia style active learning game or a presentation style session, older adults gained knowledge about safe medication practices, learned safe medication taking behaviors, and learned the importance of teaching others to do the same. These materials can be widely utilized as prevention strategies to help positively impact the issue of medication misuse in communities. This study’s findings support GROAT as an educational intervention suitable for use with universal audiences over the age of 50, as described by the Institute of Medicine’s framework for prevention programming (17). This framework specifically calls for universal prevention interventions to target outcomes which include behavioral change. While results are promising, additional research is needed to demonstrate that GROAT is equally effective among selective populations within this larger age group.

### Limitations

There are limitations of this study worth mentioning. The research design did not include a follow up survey to track sustained knowledge gains or specific behavior changes. Adding a 30 days post-intervention survey assessing the same knowledge and behavioral change for at least a subgroup of the study population would improve the study design. This is a priority for future research efforts to ascertain if the knowledge gained and positive behavioral intentions last beyond the educational event. Several approaches could be utilized to achieve this, including a phone-based follow-up or digital/mail in surveys. The study was also not designed to include a control group. A potential control group could be obtained by administering the post-survey instrument to other older individuals at the various sites who were not attending the educational sessions. This suggestion is consistent with the Whittaker study that also utilized individuals not engaged with the live educational intervention as a control group (16). To simplify the survey collection process pairing pre- and post-survey design was not attempted but would certainly strengthen the results.

There were survey implementation issues that were encountered at the programming sites. While there was considerable effort put into limiting the length of the pre- and post-surveys, facilitators and participants still found the surveys time-consuming. OSU Extension Educators planned dedicated time to administer the surveys both before the programming and again after the educational sessions to try and maximize survey responses. Individuals who arrived late to programming or did not remain to the end of the programming also contributed to missed survey opportunities or missing data responses.

## Conclusion

The growth among the older adult population in the United States is accompanied by the growing use of medications to manage the health concerns of those living longer lives. This growth in medication use increases the potential for medication misuse overall. The GenRx Older Adult Toolkit is well-suited to support the education of older adults on this topic. This study confirms through pre- and post-survey results that the GenRx Older Adult Toolkit delivered by GenRx trained OSU Extension Educators increased participants’ knowledge and favorably impacted behavioral intentions around safe medication use practices. Furthermore, the GenRx Older Adult Toolkit provides an opportunity to effectively use the education in a variety of community-based settings, thereby extending the potential reach of the education at the local level.

## Data Availability

The raw data supporting the conclusions of this article will be made available by the authors, without undue reservation.
